# Editorial: Molecular markers for pancreatic cancers: new technologies and applications in the clinical practice

**DOI:** 10.3389/fonc.2025.1651566

**Published:** 2025-07-08

**Authors:** Terence Moyana, Valeria Merz

**Affiliations:** ^1^ Diagnostic & Molecular Pathology, University of Ottawa and The Ottawa Hospital, Ottawa, ON, Canada; ^2^ Medical Oncology Unit, Santa Chiara Hospital, Trento, Italy

**Keywords:** pancreatic ductal adenocarcinoma, molecular markers, circulating biomarkers, exosomes, major vault protein, transcriptomic tools

## Introduction

This is an editorial on the Research Topic: “*Molecular Markers for Pancreatic Cancers: New Technologies and Applications in Clinical Practice*”. Experts on pancreatic cancer from various institutions contributed to the topic by addressing key points on molecular, diagnostic, predictive and prognostic markers and how they are applied in clinical practice. The editorial provides commentary and context on the articles.

Pancreatic ductal adenocarcinoma (PDAC) is by far the most dominant pancreatic malignancy, comprising approximately 90% of all the cancers therefrom (1, 2). Herein, it is used as a proxy for pancreatic cancer unless otherwise stated. It is one of the top causes of cancer-related deaths in the world with an overall 5-year survival rate of approximately 10% as highlighted by the parallelism between mortality and disease incidence (mortality-to-incidence ratio of >0.90) (3, 4). To date, the causes of PDAC are still insufficiently known, although certain risk factors have been identified e.g. smoking, obesity, genetics, longstanding chronic pancreatitis, diabetes, diet and inactivity (3, 4). The disease has an insidious onset and >80% of cases are discovered at an advanced stage when surgical resection is not feasible due to local spread or distant metastasis. Only 15-20% of patients are eligible for potentially curative surgical resection and even then most will have a recurrence, and the 5-year survival of completely resected tumors is approximately 25%. Despite advances in diagnostic techniques, perioperative management and multimodality anti-tumor therapy for advanced disease, the prognosis has not significantly improved. Since there are no current screening recommendations for PDAC for the general population (4, 5), understanding its pathogenesis and developing strategies for early diagnosis is of utmost importance.

## Traditional biomarkers

In patients suspected of having PDAC, imaging can be used for the detection of disease though it has limitations in assessing incipient/very early tumors or minimal residual disease following treatment (6). Traditional methods for investigating such patients employ serum glycoproteins such as CA 19–9, CEA and CA125 (7). Despite the routine use these biomarkers, they have significant limitations in sensitivity and specificity which render them ineffective as a screening tool in both asymptomatic and symptomatic populations (7, 8). Consequently, their clinical application is mostly confined to monitoring established disease, treatment response or recurrence. From amongst these biomarkers, CA 19–9 (also known as Sialyl Lewis-a) is historically the most widely used (7). However, it has several limitations e.g. yielding false negative results in genotypically Lewis^a-b-^ patients and false positives in patients with non-malignant conditions such as diabetes, inflammatory/obstructive biliary or respiratory disease (7, 9). Moreover, CA 19–9 is not tumor type-specific but can be overexpressed in a wide range of benign and malignant gastrointestinal and extra-gastrointestinal diseases e.g. biliary, liver, colorectum, stomach, salivary, urological, lung, breast, ovarian and thyroid neoplasms (7). Despite these limitations, CA 19–9 still finds utility in everyday clinical practice especially when used in conjunction with other parameters (7, 10). CEA is another biomarker that can be used for monitoring PDAC. It is typically produced by normal cells during embryonic development and tends to increase in inflammatory conditions or GI tumors. However, it cannot be relied upon for solitary diagnostic use since its sensitivity and specificity for early diagnosis of PDAC is even lower than that of CA 19–9 (11–13). Of the 3 cited biomarkers, CA125 is the least utilized for PDAC due to sensitivity and specificity issues (14). Hence, there is a pressing need for additional pancreatic biomarkers.

## Molecular markers

PDAC is a disease that arises from somatic and germline mutations. Work from the International Cancer Genome Consortium and The Cancer Genome Atlas has shown that the most common abnormalities involve KRAS oncogenic mutations as well as loss-of-function mutations and/or deletions of the tumour suppressor genes *TP53*, *SMAD4* and p16/*CDKN2A* (15, 16). In This Research Topic, Moretti et al. evaluated these 4 key biomarkers, exploring their potential from multiple perspectives for early disease detection and improved patient management.

KRAS has one of the highest mutation rates in PDAC with a prevalence of approximately 90% and the oncogenic driver mutations are most frequently in codons 12, 13, and 16 (17). This makes its signaling network a major target for therapeutic intervention. Hence covalent inhibitors (e.g. sotorasib) selectively targeting KRAS^G12C^ have shown promising efficacy against cancers harboring *this* mutation in clinical trials (18, 19). Whereas G12C is rare (occurs in only 1-2% of PDACs), it could be more impactful to target the more prevalent G12D and G12V mutations (occurring in approximately 40% and 30% of cases respectively). Hence clinical trials using inhibitors such as MRTX1133 are ongoing (20). P53 is a tumor suppressor gene and its mutations are also common in PDAC with a prevalence of 50-75%. Inactivation of p53, when combined with activation of KRAS has been shown to drive the development of PDAC (17). SMAD4 [also known as deleted in pancreatic cancer for (DPC 4)] is instrumental in inducing cell-cycle arrest and apoptosis, crucial mechanisms for controlling cell proliferation and eliminating damaged cells (21, 22). Not surprisingly, inactivation or dysregulation of SMAD4 is associated with PDAC progression especially in cases where the cancer has been initiated by other oncogenes like KRAS. Studies have also shown that SMAD4 mutations are associated with resistance to chemo- &/or radiotherapy, potentially serving as a biomarker for therapy stratification (23, 24). Mutations in the CKDN2A gene, which encodes the p16 tumor suppressor protein, are also associated with the development and progression of PDAC.

According to the classically held view of stepwise cancer development based on pancreatic intraepithelial neoplasia (PanIN) precursor lesions, PDAC develops through a particular sequence of mutations: KRAS, followed by CDKN2A, P53 and SMAD4 (1, 25). However, this hypothesis has been questioned because the clonally expanded precursor lesions do not always conform to these genetic alterations or mutation order. An alternative view holds that the genetic landscape holds complex and heterogenous rearrangements associated with mitotic errors consistent with punctuated equilibrium as the main evolutionary trajectory (25–27). The other somatic or germline genes involved in these mitotic errors include but are not limited to CMYC, MYB, AIB1/NCOA3, EGFR, GATA6, SWI/SNF, BRCA1/2, PALB2, ATM, CHEK2, RAD51C/D, FGFR2 and NTRK (17, 25–29).

## Circulating biomarkers

Early genomic studies on PDAC were mostly based on material derived from traditional biopsies or resection specimens (1). However, liquid biopsy techniques such as cell-free DNA (cfDNA), circulating tumor DNA (ctDNA), circulating tumor RNA (ctRNA) and circulating total nucleic acid (ctTNA) are emerging as promising avenues for improving diagnostic accuracy and treatment strategies (30, 31). They offer distinct advantages such as simplicity in sampling, minimal invasiveness and improved ability to capture intratumor heterogeneity. Additionally, ctDNA detects real-time tumor dynamics vis-à-vis archival material from a tissue biopsy or resection specimen. In This Research Topic, Arayici et al. note that detectable levels of ctDNA were associated with worse patient outcomes and overall survival. This is important because recognizing the prognostic significance of ctDNA could significantly influence treatment decisions enabling healthcare providers to tailor more personalized and effective therapeutic approaches. Nonetheless, it should be noted that the detection rate of ctDNA can be affected by multiple factors e.g. the tumor’s ability to release ctDNA into the bloodstream which in turn depends on the tumor type, dimensions, stage, vascularization, necrosis, apoptosis, metabolic activity and surrounding tissue environment. An additional critical factor is the rate at which ctDNA is cleared from the circulation. This is influenced by physiologic factors e.g. degradation by nucleases and removal by organs such as the liver and kidney. All these variables have a bearing on biomarker sensitivity and specificity in cancer detection and monitoring.

In addition to genetic mutations, epigenetic alterations such as histone modifications, chromatin accessibility and DNA methylation play a crucial role in the progression and metastasis of PDAC (32, 33). To this end, there has been a recent increase in the number of studies focusing on cfDNA analysis as epigenetic biomarkers for PDAC (34–36). In This Research Topic, Kim et al. augmented these studies by assessing the diagnostic potential of a novel DNA methylation assay based on an epigenetic-specific peptide nucleic acid (Epi-sPNA) in both tissue and plasma samples. They found that an Epi-Top pancreatic assay, along with KRAS mutations, holds potential as a biomarker for detecting PDAC from the blood.

All in all, circulating biomarker technology represents a significant development in precision medicine. However, there is currently wide variability in how ctDNA assays are developed and validated. Furthermore, since ctDNA concentrations are generally very low, the effects of variances can be amplified as the specimens are processed. Therefore, standardization is required in order to foster a consistent framework and wider clinical acceptance of these techniques (37). Variables that affect assay performance include but are not limited to: i) pre-analytical (e.g. blood collection tube, anticoagulants, blood volume, stabilization of blood cells, storage/temperature/transportation, centrifugation conditions and extraction method) and ii) analytical [e.g. DNA versus RNA-based analysis, or next generation sequencing versus polymerase chain reaction (dPCR/ddPCR), analytical sensitivity, limits of detection and specificity.

## Exosomes

Extracellular vesicles are a component of circulating biomarkers. They can be separated by size and other biophysical/biochemical properties into small and large vesicles (38, 39). The small vesicles (30–150 nm diameter) are called exosomes/nanovesicles and are secreted by multiple cell types under both physiologic and pathologic conditions. They play an important role in the transportation of biomolecules such as lipids, proteins, enzymes, mRNA, small non-coding RNA including microRNA and DNA (39–41). The exososomal cargo can modulate, instruct, and re-program adjacent target cells through autocrine or paracrine functions or on specific distant target cells.

Tumor cells face numerous challenges such as nutrient scarcity, a hypoxic microenvironment and immunologic attack, and therefore must adapt by re-wiring their signaling cascades (42). This metabolic re-programming is in part effected by the release of bioactive molecules via tumor-derived exosomes (TDEs) (39, 41, 42). Since exosomal contents can be significantly altered in PDAC, there is an emerging role for TDEs as biomarkers along the lines indicated by Zhou et al. In This Research Topic. For example, studies have shown high levels of microRNAs, epidermal growth factor receptor, CA 19-9, tumor-associated mucins, KRAS mutations and claudin 1 in TDEs from PDAC (43, 44).

Lastly, with their favorable biodistribution and biocompatibility, exosomes have recently garnered considerable attention as potential vehicles for drug delivery in PDAC treatment. Drug delivery systems such as engineered exosomes (iExosomes) can be used to target e.g. KRAS mutations such as KRAS^G12D^ which are prevalent in PDAC (45–47). Clinical trials using exosomes as drug carriers are now underway or have been completed (48). Zhou et al.’s bibliometric analysis serves to illustrate the growing interest in the role of exosomes in the biology of PDAC.

## Major vault protein

Major vault protein (MVP), also known as the drug resistance-related protein, is a major component of multi-subunit ribonucleoprotein particles (also known as vaults) which are involved in nuclear-cytoplasmic transport (49, 50). Elevated expression of MVP has been shown to promote cancer progression in various malignancies such as breast, prostate and liver (51–53). In This Research Topic, Wu et al. demonstrated a markedly increased expression of MVP in pancreatic cancer which significantly correlated with an adverse prognosis. In a series of related analyses, they further confirmed its potential as a diagnostic and prognostic indicator for PDAC, which is in accord with studies of other cancer types (51–53). In line with the observations of Kim et al., they also found that aberrant methylation may play a role in PDAC initiation and progression. In addition, they observed a negative correlation between MVP expression and the IC50 of oxaliplatin, which suggests a potential avenue for optimizing oxaliplatin administration in PDAC patients.

## Transcriptomic tools for predicting chemotherapy response

One of the main treatment options for patients with advanced PDAC is chemotherapy which can be either single agent or combined therapies (54, 55). With regard to monotherapies, gemcitabine is generally regarded as one of the most effective for PDAC, and is often deployed in patients unfit for more aggressive treatments (56). Among the combined regimens, modified FOLFIRINOX (mFFX) comprised of 5-fluorouracil, leucovorin, irinotecan and oxaliplatin appears to be a promising approach. However, the effectiveness of mFFX is limited by drug toxicities such as neutropenia, thrombocytopenia, diarrhea and sensory neuropathy. Consequently, the use of mFFX is to a considerable extent contingent upon the patient’s performance. Ideally it would be advantageous to assess the effects of each drug within the mFFX regimen with the objective of minimizing unnecessary toxicity but without compromising clinical benefits. In This Research Topic, Fraunhoffer et al. used cell lines and organoids to develop transcriptomic signatures which define sensitivity for each of these drugs in order to capture the biologic components responsible for the response to each drug. This can be used to modify/rationalize the mFFX regimen and help to avoid unnecessary toxic effects. Concurrently, transcriptomic signature studies are also being conducted on gemcitabine-based regimens (e.g. gemcitabine plus capecitabine or gemcitabine plus nab-paclitaxel) to provide more options for the therapeutic landscape (56).

## Chronic hepatitis B infection

The association between chronic hepatitis B virus (HBV) infection and hepatocellular carcinoma is well-known (57). Epidemiologic studies suggest that there may also be an association between HBV and PDAC (58–60). In support of this notion is the fact that both liver and pancreas are embryologically of foregut endodermal origin and share a similar blood supply. Furthermore, HBV DNA has been isolated from pancreatic tissue of individuals with PDAC (58, 61). In This Research Topic, Long et al. found that past exposure to HBV infection was associated with better overall survival in patients with metastatic PDAC. However, other studies have produced discrepant results (58–60). While such differences could be due to patient samples, further studies are required to reconcile these differences and/or determine whether HBV-associated PDAC has distinctive features.

## Conclusion

PDAC remains a formidable malignancy with a poor prognosis and is projected to be the second leading cause of cancer death in the not so distant future. While traditional tissue biopsies and serum glycoproteins are still useful, there is clearly a need for newer approaches, emphasizing effective biomarkers to enhance disease detection, treatment selection and patient outcomes. Recent advances in molecular profiling have identified potential biomarkers for early diagnosis, targeted therapies and prognosis. Molecular markers such as KRAS, TP53, SMAD4 and p16/CDKN2A and circulating biomarkers including exosomes show promise in enhancing diagnostic accuracy and prognostic evaluations. Furthermore, genetic alterations, for example KRAS^G12C,G12D,& G12V^ and BRCA 1/2 are emerging as predictive biomarkers for targeted treatments including PARP inhibitors and immunotherapy. Using diagnostic algorithms and machine learning, these biomarkers can be incorporated into datasets for more precise disease management. All in all, a paradigm shift is underway in molecular testing for pancreatic cancer.
